# Diffuse Large B-cell Lymphoma Causing Central Airway Obstruction: A Case Report

**DOI:** 10.7759/cureus.77507

**Published:** 2025-01-15

**Authors:** Rabi Shrestha, Subekshya Khadka, Hollie Saunders, Scott Helgeson

**Affiliations:** 1 Internal Medicine, Crestwood Medical Center, Huntsville, USA; 2 Pulmonary and Critical Care, Mayo Clinic in Florida, Jacksonville, USA; 3 Critical Care and Pulmonology, Mayo Clinic in Florida, Jacksonville, USA

**Keywords:** central airway obstruction, dlbcl, lymphadenopathy, nhl, non-hodgkin lymphoma, pmbcl, primary diffuse large b-cell lymphoma, primary mediastinal large b-cell lymphoma

## Abstract

Primary diffuse large B-cell lymphoma (DLBCL) is the most common type of non-Hodgkin lymphoma (NHL) globally, while primary mediastinal B-cell lymphoma (PMBCL) is a rare subtype that may lead to central airway obstruction by compressing or invading the airways. Prompt management of airway obstruction is crucial, as severe cases can be life-threatening if left untreated.

This study reports the case of a 27-year-old man with chronic cough, weight loss, and computed tomography (CT) of the chest revealing a large anterior mediastinal tumor with an extension into the neck and left lung causing airway compression and complete left lung collapse who was found to have PMBCL. The aim of this case report is to show that, in some cases of malignant central airway obstruction, the treatment approach should be based primarily on treating the underlying cause instead of procedural intervention, depending on clinical presentation and history of response to therapy. We highlight the treatment strategy of primary therapy for DLBCL to address central airway obstruction rather than surgical or bronchoscopic intervention.

## Introduction

Primary mediastinal B-cell lymphoma (PMBCL) has an annual incidence of 0.4 per million, and with appropriate treatment, the five-year survival rate is 80-95% [1]. More than two-thirds of PMBCL cases present with a large anterior mediastinal mass, which may result in local compression, resulting in dyspnea, cough, dysphagia, compromised airways or great vessels, and superior vena cava obstruction [1,2]. Primary mediastinal B-cell lymphoma primarily occurs in young females and arises in the mediastinum, frequently exhibiting local invasion. Historically, R-CHOP therapy, short for rituximab, cyclophosphamide, doxorubicin, vincristine, and prednisolone, has been the first-line treatment, followed by involved-site radiotherapy (ISRT). However, treatment approaches differ, with a growing preference for avoiding early radiotherapy and using dose-intensive regimens like drug-adjusted etoposide phosphate, prednisone, vincristine sulfate (Oncovin), cyclophosphamide, doxorubicin hydrochloride (hydroxydaunorubicin), and rituximab (DA-EPOCH-R) in younger, healthy patients [1,2].

We describe a case of PMBCL, with large mediastinal lymphadenopathy, in which the patient had an almost complete obstruction of the left mainstem bronchus. We highlight that therapy was focused on the underlying pathology without the need for bronchoscopic or surgical intervention to treat the central airway obstruction.

## Case presentation

**Figure 1:**
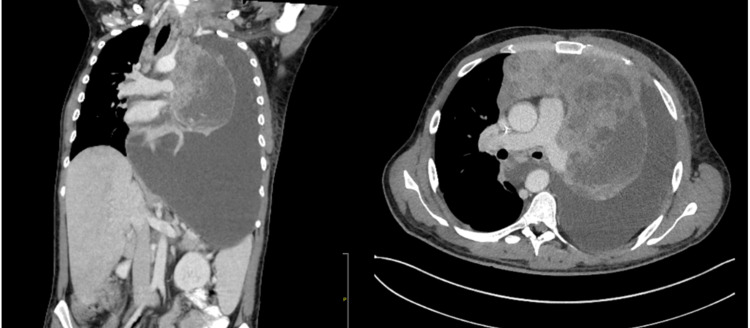
Axial and sagittal computed tomography images at the time of presentation of the large anterior mediastinal mass with complete left lung atelectasis and associated large left-sided pleural effusion

A 27-year-old male patient of Hispanic origin, with a 10-pack-year smoking history and no other medical comorbidities, presented with three months of cough, dyspnea, left-sided chest heaviness, and weight loss. Laboratory testing was significant for a hemoglobin of 8.9 g/dl (13.2-16.6 g/dl). All other laboratory testing was within normal limits. A computed tomography (CT) of the chest and neck revealed a conglomerate of anterior mediastinal and left lower cervical lymph nodes measuring up to 18x8.7 cm, along with complete left lung collapse with invasion from the mass and large left pleural effusion (Figure 1). Following these CT scans, he was admitted to the hospital for treatment and further workup.

**Figure 2:**
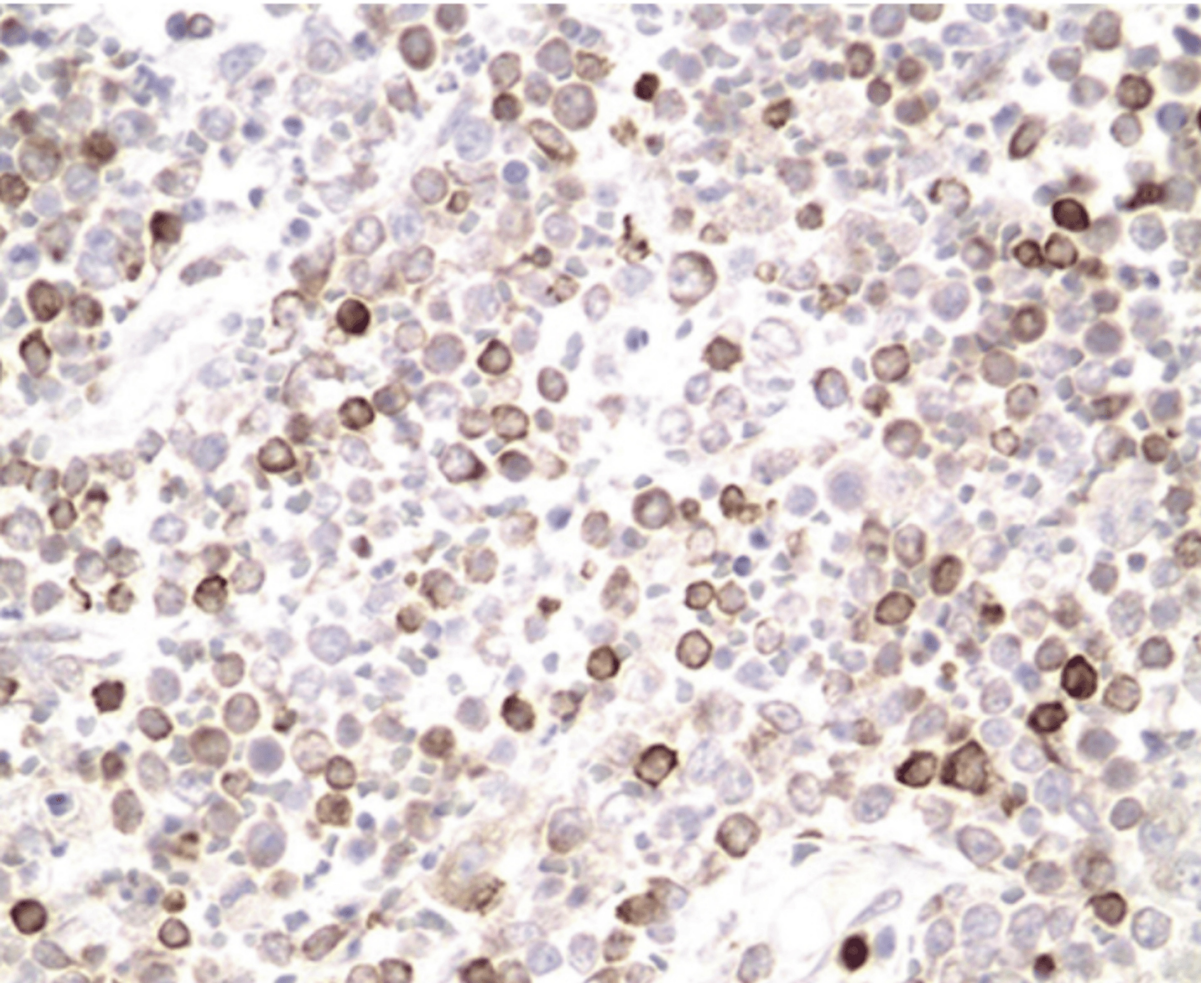
Diffuse large B-cell lymphoma mediastinal tissue membrane staining for BCL-2 (BCL-2/peroxidase immunostaining)

**Figure 3:**
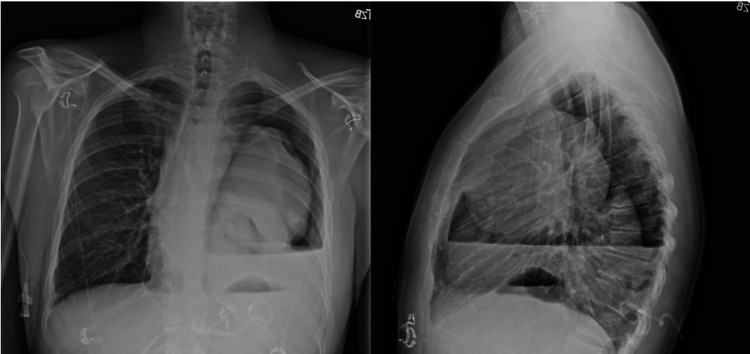
Anteroposterior and lateral chest X-ray images of the chest following drainage of the pleural effusion with persistent left lung atelectasis and large ex vacuo pneumothorax

A CT-guided fine needle biopsy of the mediastinal mass was diagnostic of PMBCL with immunohistochemistry being positive for CD20, PAX5, BCL2, BCL6, MUM1, and C-MYC (Figure 2). A bone marrow biopsy revealed no abnormality in his cell lines. A chest tube was placed for the large pleural effusion with cumulative drainage of 4 liters of fluid. Pleural fluid analysis revealed 83% lymphocytes, lactate dehydrogenase of 583 U/L, and protein of 2.3 g/dL. Cytology of the pleural fluid was negative for malignancy. Following drainage of the pleural effusion, a chest x-ray revealed a significant ex vacuo pneumothorax and sustained entire left lung atelectasis (Figure 3). Pulmonology was consulted to discuss various interventions for his complex central airway obstruction. Due to minimal respiratory symptoms and no need for supplemental oxygen, bronchoscopic intervention was deferred, and therapy was focused on treating the lymphoma. The patient was discharged from the hospital after seven days with no respiratory symptoms.

**Figure 4:**
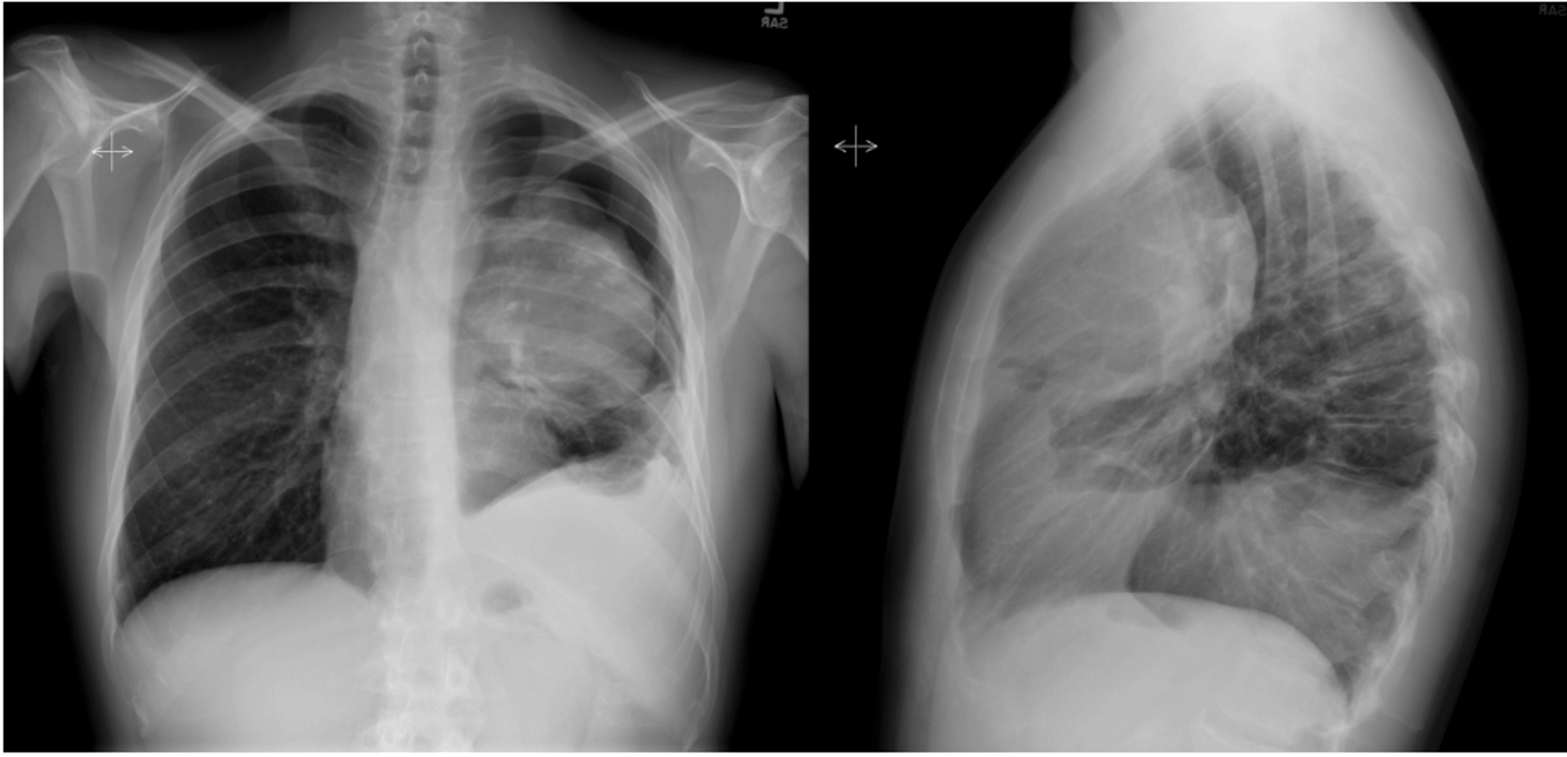
Anteroposterior and lateral chest X-ray four months after initiating treatment with improved aeration of the left lower lobe and lingula. Persistent atelectasis of the left upper lobe is noted.

**Figure 5:**
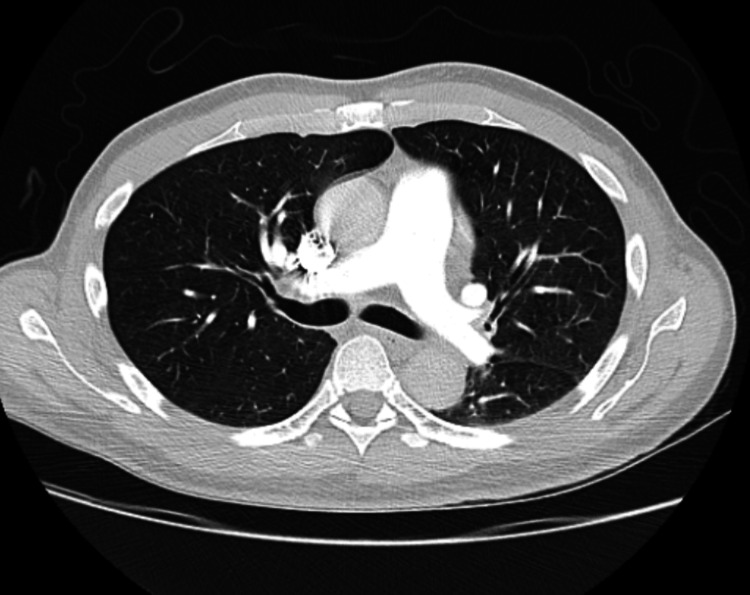
Axial computed tomography of the chest after completion of treatment with re-expansion of the left upper lobe

Four months after chemotherapy, imaging revealed a reduction in the mass’s size with improved aeration of the left lung (Figure 4). The patient responded favorably to the six cycles of chemotherapy and achieved complete remission as evidenced by CT scans showing ongoing improvement in the size of the mass and expansion of the left lung (Figure 5).

## Discussion

Primary mediastinal B-cell lymphoma is rare and accounts for 2%-4% and 7% of non-Hodgkin’s and diffuse large B-cell lymphoma (DLBCL), respectively [1]. Chemotherapy is the most common initial treatment for DLBCL, with disease remission occurring in 50% to 60% of patients [3]. Overall survival and progression-free survival at five years have been found to be 75% and 69%, respectively, or 81% and 73%, respectively, if aged <40 years [4].

Central airway obstruction is any process resulting in the narrowing of the trachea, mainstem bronchi, or bronchus intermedius on imaging or by direct visualization [5]. Central airway obstruction can be due to intrinsic (airway pathology) or extrinsic causes (external compression) and can be further divided into malignant and non-malignant causes [6]. Malignant causes include metastatic cancer, bronchogenic cancer, as well as mediastinal lymphadenopathy, including lymphoma and thymoma [6]. Three categories of non-malignant causes have been identified: previous airway trauma (post endotracheal tube, post tracheostomy, anastomotic stricture), medical conditions (Wegener's granulomatosis, sarcoidosis, and relapsing polychondritis), and other causes (idiopathic, tracheomalacia, congenital webs, carcinoid) [6]. 

Multiple methods are employed to treat central airway obstruction, including airway dilation, thermal ablative techniques, tracheobronchial stenting, cryotherapy, and radiation [6,7]. These interventions come with risks, including bleeding, airway perforation, hypoxemia, fistula formation, strictures, and air embolism [6]. Rates of these complications are relatively low and range from 0.1% to 11%, with mortality typically between 0% and 0.1% [8]. However, in cases of stenting for malignant central airway obstruction, the complication rate is reported to be 23% to 34%. These complications include infection, mucous plugging, stent migration, and stent fracture. In addition, clinically significant improvement in dyspnea and health-related quality of life was only 48% and 42%, respectively [9].

Planned intervention should be weighed against anticipated risks and the chance of procedural success. Larouche et al. observed only a 3.6% risk of relapse occurring five years or later following diffuse large B-cell lymphoma (DLBCL) diagnosis in 1,492 patients [10]. Cases have reported the use of airway stenting for central airway obstruction from lymphoma have shown varied results [11,12]. In a case report by Chawla et al., a 45-year-old man who had mediastinal non-Hodgkin's lymphoma complicated by central airway obstruction and respiratory failure underwent a Y-shaped self-expandable metallic stent placement in conjunction with chemotherapy [11]. Chest radiography shortly after the second cycle of therapy demonstrated a reduction in the size of the tumor, making it possible to remove the stent [11]. Choo et al. reported a patient with Burkitt’s lymphoma with tracheal and left mainstem bronchi compression. The placement of a Y-shaped silicone stent did not relieve the obstruction, and she required venovenous extracorporeal membrane oxygenation (VVECMO). She displayed a rapid response to chemotherapy and was able to be weaned from VVECMO after three days and extubated after six days [12]. Alnahas et al. described a case of thyroid lymphoma complicated by central airway obstruction leading to respiratory failure and the need for invasive mechanical ventilation [13]. The patient was deemed to not be a candidate for any intervention and was treated with salvage chemotherapy. This therapy led to a significant decrease in lymphoma and a reversal of airway obstruction within a few days of initiation [13].

Our patient had a case of complete left mainstem obstruction due to a large and invading anterior mediastinal mass with complete left lung entrapment and atelectasis. The unique aspect of the case was the unusual presentation of this malignancy and mild respiratory symptoms exhibited by the patient. Often, in cases such as these, invasive measures would be considered to allow for lung re-expansion. However, knowing the good response rates of DLBCL to treatment and the risks of procedural intervention, we were able to focus entirely on cancer-directed therapy with resultant re-expansion of the lung without the need for bronchoscopy or surgery.

## Conclusions

This case demonstrates how, in certain cases of malignant central airway obstruction, treatment can be focused on therapy for the underlying pathology without the need for procedural or surgical intervention. This should be taken into consideration in patients with a well-compensated respiratory status and a malignancy that has historically responded well to therapy.
